# SOMAScience: A Novel Platform for Multidimensional, Longitudinal Pain Assessment

**DOI:** 10.2196/47177

**Published:** 2024-01-12

**Authors:** Chloe Zimmerman Gunsilius, Joseph Heffner, Sienna Bruinsma, Madison Corinha, Maria Cortinez, Hadley Dalton, Ellen Duong, Joshua Lu, Aisulu Omar, Lucy Long Whittington Owen, Bradford Nazario Roarr, Kevin Tang, Frederike H Petzschner

**Affiliations:** 1 Robert J. and Nancy D. Carney Institute for Brain Science Brown University Providence, RI United States; 2 Neuroscience Graduate Program Department of Neuroscience Brown University Providence, RI United States; 3 Warren Alpert Medical School Brown University Providence, RI United States; 4 Department of Cognitive, Linguistic, and Psychological Sciences Brown University Providence, RI United States; 5 Department of Neuroscience Brown University Providence, RI United States; 6 Center for Computation and Visualization Brown University Providence, RI United States; 7 Industrial Design Rhode Island School of Design Providence, RI United States; 8 Department of Psychiatry and Human Behavior Brown University Providence, RI United States; 9 Center for Digital Health Brown University, Lifespan Providence, RI United States

**Keywords:** acute pain, acute-chronic pain transition, chronic pain, clinical outcome measurement, digital health, ecological momentary assessment, EMA, ESM, experience sampling methodology, mHealth, mobile health, pain management, pain self-management, patient reported outcomes, smartphone app

## Abstract

Chronic pain is one of the most significant health issues in the United States, affecting more than 20% of the population. Despite its contribution to the increasing health crisis, reliable predictors of disease development, progression, or treatment outcomes are lacking. Self-report remains the most effective way to assess pain, but measures are often acquired in sparse settings over short time windows, limiting their predictive ability. In this paper, we present a new mobile health platform called SOMA*Science*. SOMA*Science* serves as an easy-to-use research tool for scientists and clinicians, enabling the collection of large-scale pain datasets in single- and multicenter studies by facilitating the acquisition, transfer, and analysis of longitudinal, multidimensional, self-report pain data. Data acquisition for SOMA*Science* is done through a user-friendly smartphone app, SOMA, that uses experience sampling methodology to capture momentary and daily assessments of pain intensity, unpleasantness, interference, location, mood, activities, and predictions about the next day that provide personal insights into daily pain dynamics. The visualization of data and its trends over time is meant to empower individual users’ self-management of their pain. This paper outlines the scientific, clinical, technological, and user considerations involved in the development of SOMA*Science* and how it can be used in clinical studies or for pain self-management purposes. Our goal is for SOMA*Science* to provide a much-needed platform for individual users to gain insight into the multidimensional features of their pain while lowering the barrier for researchers and clinicians to obtain the type of pain data that will ultimately lead to improved prevention, diagnosis, and treatment of chronic pain.

## Introduction

More of us are in chronic pain than you might think—20% of adults in the United States reported pain on most or several days in 2019 [1]. This presents a substantial burden on society, costing up to US $635 billion annually [2]. In 2016, chronic back and neck pain alone accounted for the highest amount of US health care spending across 154 conditions, including diabetes and heart disease [3]. Moreover, pain is the leading cause of health care use across all illnesses [4-6]. Against this backdrop, it has never been more important to develop accurate pain symptom assessment and prediction methods to help patients, caregivers, and other stakeholders make informed decisions about treatment and care.

Accurately measuring pain is crucial for predicting an individual’s pain trajectory [7]. Methods to identify objective biomarkers of pain intensity [8-10] are still in their infancy and have yet to be proven effective in predicting future self-reported pain [11]. To date, the most common way to assess if someone is in pain is to simply ask them [12]. Typically, this is done using an 11-point pain intensity scale, where individuals are asked to rate their pain from 0 (no pain) to 10 (worst pain imaginable) [12-14]. Since the 1980s, this simple pain intensity scale has played a significant role in the clinical assessment of pain by enabling defined targets for pain management and the dosing of pain-relieving medications [15,16]. If a person consistently reports pain intensity as more than 3 out of 10 for more than 3 months, the scale becomes part of the diagnostic criteria for chronic pain [17]. For patients, self-reporting their pain on the scale can validate and quantify their pain experience, leading to improved shared decision-making and enhanced communication with health care providers [18].

Despite its ease of use, the pain intensity scale has not led to significant advancements in pain management or patient satisfaction [18-23]. When measured in medical settings, people tend to over- or underreport their pain intensity depending on difficulties with recall, expected treatments, care standards, or other subjective factors such as mood [24,25]. Additionally, regulatory approval for pharmaceutical companies to promote “titration to effect” practices, whereby physicians were encouraged to increase opioid doses to achieve continued reductions on the pain intensity scale, contributed to opioid overprescribing that fueled the opioid epidemic [23,25]. These limitations highlight the need for more nuanced self-report measures of pain.

In recent years, clinical approaches to pain have sought to better assess the multidimensional experience of pain from a biopsychosocial perspective [12,26]. Multidisciplinary and individualized assessment and treatment of pain with both pharmacologic and nonpharmacologic interventions is now considered the ideal way to treat both acute and chronic pain [27]. To overcome the limitations of the unidimensional pain intensity scale, expert panelists at the Food and Drug Administration (FDA) and National Institutes of Health (NIH) have established “core outcome sets” of multidimensional questionnaires for use in research studies [13,28,29]. Dimensions assessed include pain, unpleasantness, interference, and impacts on mood and activity [25,30]. However, completing multiple questionnaires is time-consuming, and therefore not ideal for daily longitudinal studies. As a result, a large barrier remains in the ability of researchers to collect comprehensive, multidisciplinary pain data sets [31]. It is therefore imperative in pain research to implement a reliable method for multidimensional pain measurements that accurately captures the most important dimensions of pain symptoms and treatments as they evolve over time in the context of people’s daily lives.

A comprehensive approach to pain assessment necessitates the acquisition of both deep and wide pain data. Deep data involve in-depth evaluations of the multidimensional aspects of pain within individuals over extended periods, while wide data refer to data sets that encompass a large number of individuals across different demographic factors like age, geographic location, race, ethnicity, and socioeconomic status, as well as across different pain diagnoses (eg, arthritis, fibromyalgia, and postsurgical pain). The acquisition of deep data enables a deeper understanding of the mechanisms that trigger and sustain pain in individuals, while wide data provide the foundation for generalizing findings and developing biomarkers for pain persistence or recovery. Smartphone apps can provide large-scale platforms for data collection while also helping users track their daily symptom experience [32]. Such digital tools provide a promising solution for acquiring deep and wide data sets that enable new behavioral and scientific insights into the dynamics and evolution of pain.

Here, we introduce a novel mobile health (mHealth) platform for longitudinal pain assessment, called SOMA*Science*. This platform has been conceptualized to comprehensively capture multiple facets of pain through its smartphone app, SOMA. Unlike conventional pain assessment tools that focus primarily on daily pain intensity, SOMA offers an enriched multidimensional pain assessment. This includes factors like daily pain intensity, unpleasantness, interference, mood, expectations, and activities, based on experience sampling methodologies (ESMs) [33]. Our choice of measures is in accordance with the current Initiative on Methods, Measurement, and Pain Assessment in Clinical Trials (IMMPACT) recommendations for pain assessment [28,34]. In addition, the design and validation of the SOMA app have been executed following the guidelines set forth by the American Psychiatric Association (APA) [35,36].

SOMA was designed with users in mind, offering a free and user-friendly tracking feature that facilitates real-time tracking of pain, medications, and treatment regimens. A “Trends” section distills the multidimensional data to visualize pain trends. This feature aids users in self-managing their pain, recognizing patterns, and discerning between effective pain management and areas needing improvement. With these insights, users can communicate more efficiently with their health care providers, positioning SOMA as a valuable “companion” tool to be used alongside standard medical advice.

SOMA*Science* has been engineered to provide researchers with the ability to carry out independent single- and multicenter studies. Its pain assessments can be used as end points in research studies and within clinical practice. To support expansive research initiatives, the SOMA*Science* platform ensures seamless transfer of app-sourced data to researchers and affiliated institutions and offers open-source code to streamline data preprocessing.

Contemporary pain research standards underscore the significance of multidimensional pain assessments through established batteries of pain questionnaires [13,29]. However, we identified a scarcity of available tools that effectively capture such assessments in a format meaningful for researchers aiming to develop better measures of pain outcomes or symptom burden over time. While there are smartphone apps, like Manage My Pain and Pain Scale-Pain Tracker App, they primarily cater to users as self-management tools [37,38]. The foundational architecture of such apps and the nature of the data they procure do not typically align with the specifications researchers need for conducting or analyzing comprehensive, large-scale studies that meaningfully contribute to pain research. A further limitation is the proprietary nature of many of these apps, which can impede data quality, accessibility, and transparency.

To our knowledge, no other tools currently exist that are specifically designed to capture multidimensional and longitudinal pain metrics in compliance with the recommended standards and are simultaneously tailored for extensive open-source academic research. This distinctiveness sets SOMA*Science* apart as an unparalleled mHealth platform.

In this paper, we discuss the choices and technological considerations for the development of SOMA*Science* as well as the scientific rationale behind the selection of measures. Our aim is to outline how SOMA*Science* can be used by scientists and institutions to acquire large-scale, longitudinal, multidimensional pain data in single and multicenter studies in order to gain new insights into pain that will benefit patients.

## Section 1: SOMA*Science* Platform

### Overview

SOMA*Science* aims to address the current shortage of platforms for acquiring both deep (multidimensional and longitudinal) and wide (cross-spectrum and large-scale) pain data to create novel insights into the dynamics and evolution of acute and chronic pain. The term SOMA stems from the Greek word σόμα (meaning body or entire person), signifying that it takes a holistic approach to pain.

**Figure 1:**
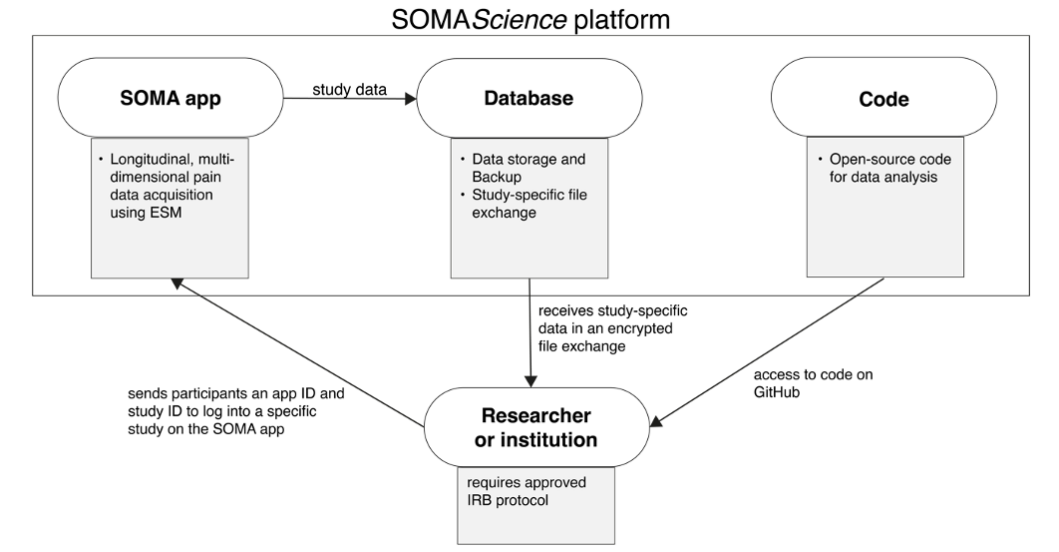
Schematic overview of the SOMA*Science* platform. Data will be acquired using the SOMA app, a user-friendly smartphone app available on Google Play and the Apple App Store. Encrypted data are sent to our application programming interface (currently located at Brown University) and shared in a study-specific manner with individual researchers and institutions. To facilitate data analysis, we created a GitHub repository where researchers can download, modify, or even create new versions of our template scripts for data preprocessing and certain analysis techniques through GitHub. ESM: experience sampling methodology; IRB: institutional review board.

SOMA*Science* represents the combination of 3 branches: the SOMA smartphone app used for data acquisition, an application programming interface for data transfer, and open-source analysis code distributed through GitHub [39] (Figure 1).

**Figure 2:**
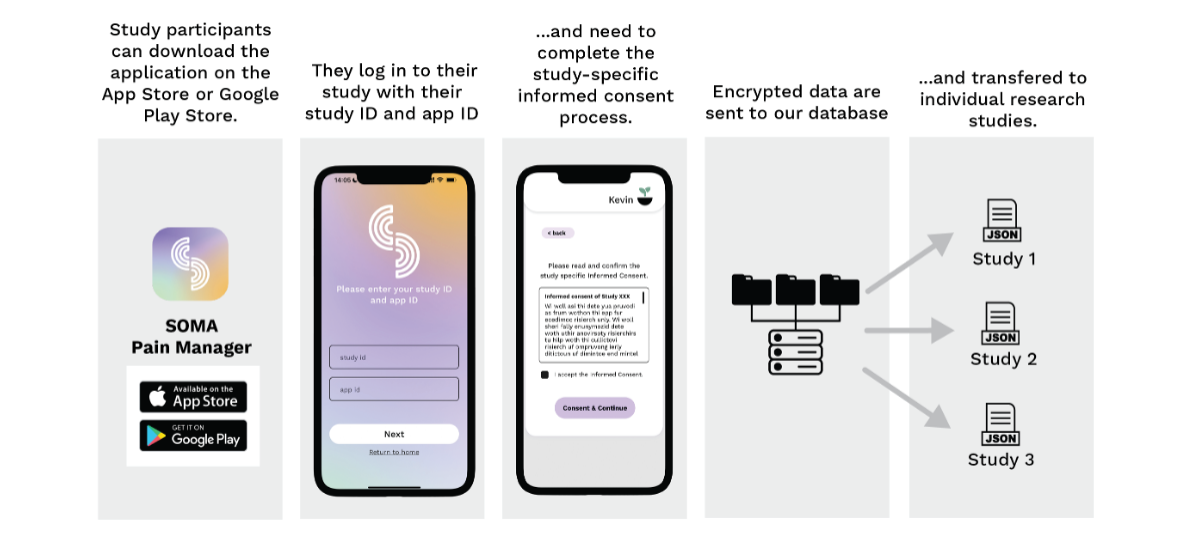
Schematic overview of study enrollment on the SOMA app. Study participants can download and register on the SOMA app directly using the links to Google Play or the Apple App Store or by searching for “SOMA Pain Manager.” Inside the main menu of the app, they can sign into a specific research study using a pre-sent study ID and App ID. They are shown a copy of their institutional review board–approved study-specific informed consent form for their records. Encrypted research data on SOMA will be sent to our database and transferred to the researchers of each individual study.

To request to run a study through the platform, researchers need to submit a research inquiry detailing the study purpose on the SOMA website [40]. Data for SOMA*Science* are acquired through the SOMA app, which is freely available on Google Play [41] and the Apple App Store [42] and can be found by searching for “SOMA Pain Manager.” Anyone is able to download and use the app, regardless of whether they are participating in a research study. For associating people’s app data with a specific study, the researcher will be assigned a unique study ID (1 per study) and a list of individual app IDs (1 per expected participant), which need to be sent to the study participants. Participants can then install and register on the SOMA app and enroll in a specific study using the study ID and unique app ID (instruction videos on [43]; Figure 2). This use of study and app IDs allows the assignment of individual participants’ data to single and multicenter studies.

Upon enrollment, participants will be sent regular reminders through the app to fill out short pain surveys (details about the ESM and data content are in the following sections). At the time of publication, assessments are restricted to the features listed below. Future releases may offer the option to request additional features and questions. After each assessment, encrypted data are transferred to our application programming interface, stored on an actively managed secure database (see “Maximizing Privacy and Security” in Multimedia Appendix 1), and then shared with researchers from individual studies (Figures 1 and 2).

### Implementation of APA Guidelines in SOMA

The APA’s app evaluation model stands as a notable benchmark for evaluating the suitability of health-related smartphone apps intended for patient populations [35]. It delineates 5 pivotal criteria to assess apps: accessibility, privacy and security, clinical foundation, engagement style, and therapeutic goal [44]. To keep pace with the rapidly advancing field of health apps, the system is regularly updated and refined [45]. Stemming from this APA model is a comprehensive database [46], which facilitates app evaluations across the 5 core domains [47]. Such initiatives are crucial in establishing public-facing, user-friendly standards for health apps and ensuring the development of safe and efficacious apps that benefit users [48-50].

In the development of SOMA*Science*, we have deeply integrated the cardinal principles of the APA model. Recognizing the emphasis that this model (and other akin evaluation frameworks) places on robust privacy, security, usability, and clinical foundations, we meticulously factored in specific technological elements during the app’s creation [50]. For readers interested in the technological nuances and our dedicated approaches to privacy and security aligned with the APA guidelines, we direct you to Multimedia Appendix 1 [51-60].

### Development of SOMA*Science*

A multidisciplinary team at the Psychiatry, Embodiment, and Computation Lab at Brown University, comprising academic neuroscientists, psychologists, pain physicians, software engineers, and designers, collaboratively conceptualized and initialized the early design and features of the SOMA app. The SOMA*Science* platform is supported by the Brainstorm program at the Carney Institute for Brain Science at Brown University, a new program to accelerate the translation of computational brain science into real-world applications that benefit patients, the scientific community, and society as a whole.

### Incorporation of Patient and User Feedback

Any app meant for long-term use must provide a simple, user-friendly interface tailored to its target audience. With this in mind, we actively sought initial feedback from individuals experiencing chronic pain to shape our app’s delivery structure. Through comprehensive one-on-one Zoom (Zoom Video Communications, Inc) discussions with a select patient group (n=4), representing a diverse pain spectrum including migraines, postsurgical musculoskeletal pain, cancer pain, and inflammatory bowel disease, we gleaned insights into our early app prototypes. These discussions involved a detailed walkthrough of each interface, where patients aired their thoughts in a guided interview.

Drawing from this feedback, we refined the “Check-In” interface’s design and flow. A common sentiment among initial users was the importance of daily tracking of pain and mood fluctuations in tandem with daily activities. To accommodate this, we introduced the “Trends” screen, a visual tool designed not only to foster self-management and a deeper understanding of pain dynamics but also to facilitate effective communication with health care providers. To further enhance the app’s utility, we incorporated screens to monitor various treatment modalities and transitioned the mood and emotion tracking interfaces to use intuitive visual analog scales (VASs) in lieu of a 2D rating system.

Responding to the patient’s desire for a more personalized experience, we introduced an interactive chatbot during the onboarding phase. This chatbot briefly engages users, gathering foundational demographic details and an introductory snapshot of their pain experiences. Existing studies vouch for the efficacy of chatbots in extending support to people with chronic pain [61], making this an evolving component with forthcoming features in SOMA that focus on pain interventions.

Our iterative refinement strategy incorporated a beta-testing phase. Initially, 30 internal testers actively engaged with the app daily across a gamut of devices and operating platforms, enabling us to identify and correct technical glitches and enhance the user experience, especially regarding the “Trends” data visualization. To expand our feedback, SOMA was then shared with a larger patient interest group (over 250 people with chronic pain), leading to critical refinements and the inclusion of user-suggested enhancements. This ongoing feedback mechanism ensures the continuous improvement and evolution of SOMA.

## Section 2: Pain Data Acquisition Through SOMA*Science*

### Deep Data Acquisition Using ESMs

#### Overview

The SOMA app uses ESMs to gather multidimensional and longitudinal pain data for SOMA*Science*. ESMs, also known as ecological momentary assessments, provide real-time, self-report data about individuals’ thoughts, feelings, and experiences (eg, “How do you feel right now?”) in the context of people’s daily lives [33]. Previous ESM studies on pain have shown high completion rates (>85%) and demonstrated the feasibility of using these daily self-reports for pain [62], in line with findings about the high completion rates of mobile-delivered ESM studies in general [63].

ESMs have several benefits over traditional self-report measures. First, they offer real-time data that are less prone to recall bias, allowing for the capture of critical experiences that might be missed by retrospective long-term self-report measures [64-67]. Second, ESMs can capture contextual information about an individual’s thoughts and experiences, such as knowing what activities a person engaged in when they experienced pain [68]. Thus, they provide insight into the longitudinal dynamics of multidimensional aspects of pain in people’s natural ecological environments across time and context [69,70]. This enables the generation of rich data sets that could be used to identify candidate behavioral “biomarkers” or “assays” that predict transitions in disease states based on self-report alone [68,71]. For example, there is preliminary evidence that longitudinal measures of pain can predict acute pain state transitions after surgery [72] and identify treatment response time courses in patients with chronic pain [73].

#### Momentary, Situational, Retrospective, and Prospective Assessments in SOMA*Science*

**Figure 3:**
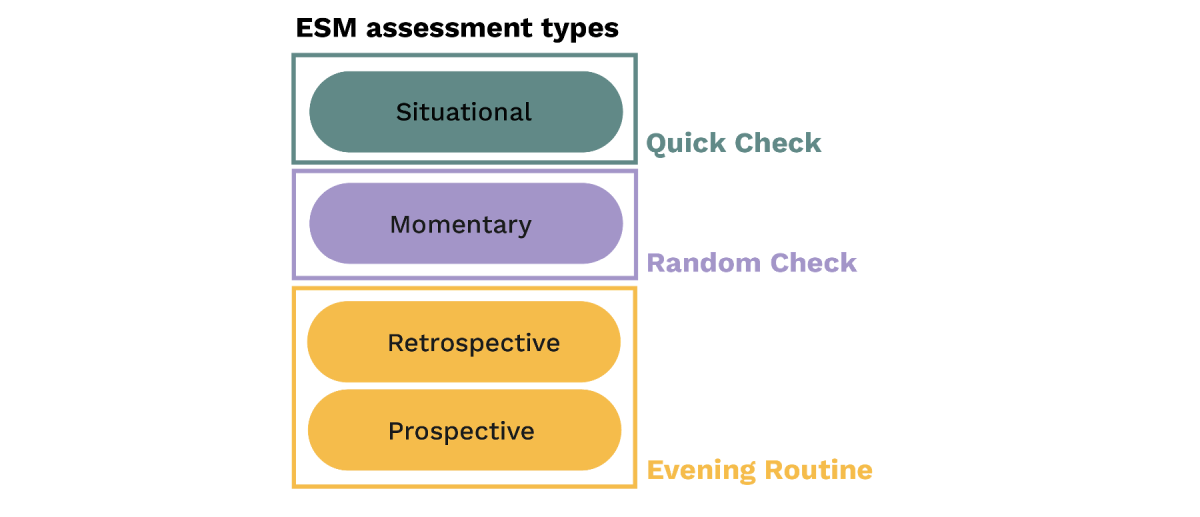
ESM assessment types in SOMA*Science*. Users complete 3 main types of daily check-ins per day (Quick Check, Random Check, and Evening Routine) that assess different domains of their daily experiences. ESM: experience sampling methodology.

One limitation of existing ESM studies is that they typically solicit several short, momentary reports throughout the day [74]. While this approach reduces bias in pain reports resulting from memory recall or pain beliefs, it may still miss important short-term pain dynamics, such as flare-ups, and fail to assess the role of expectations in the development and treatment of pain [75]. To address this limitation, SOMA*Science* uses a multifaceted approach, which includes 4 daily assessment types on the SOMA app: momentary assessments (called random check-ins), voluntary self-initiated entries (called quick check-ins), and both retrospective and prospective assessments or coverage assessments [75] (which are both part of an evening routine at the end of the day; Figure 3).

Random and quick check-ins capture various aspects of mood, activities, pain, and pain location and can be completed in less than 30 seconds. Quick check-ins can be performed at any time, for example, during or shortly after a flare-up. Random check-ins reflect classical ESM assessments and only occur during randomly selected moments within a specific time window (eg, 3 checks per day between 8 AM and 6 PM). Users receive notifications on their phone when the random check-in is available and have the option to snooze the notification for a predefined time window (eg, a maximum of 60 minutes).

The evening routine assesses recall of pain, mood, activities, and any pain-related treatments over the past day (retrospective), as well as predictions of pain, mood, and activities for the next day (prospective). This routine is available during a prolonged, preset, but fixed time window at night (eg, 6 PM-11 PM) to promote habit formation that increases the likelihood of long-term app use. It takes less than 3 minutes to complete.

#### Longitudinal Assessments with SOMA*Science*

Pain is inherently dynamic, fluctuating not just daily but hourly and even on a minute-to-minute scale, even without changes in physiological markers [18,47,62]. Traditional methods, which measure pain intensity sporadically during clinical trials or medical visits, might not capture a patient’s holistic pain experience due to their limited assessment windows.

While some studies aim for more granular pain assessments, they often focus on brief periods. A systematic review found the median duration for ESM studies in pain to be 14 days and a mere 7 days for general mHealth ESM studies [40,61]. Such short durations can overlook pivotal phases, like the transition from acute to chronic pain over 3-6 months. Consequently, the role of self-reported pain dynamics in acute pain recovery or its evolution to chronic pain remains ambiguous.

The SOMA app is designed to bridge this gap. Its check-ins are concise, using straightforward design principles for ease of use. The chosen metrics cater to diverse pain experiences, facilitating collaboration and data sharing among researchers. Moreover, the app’s “Trends” feature empowers users to track their pain, treatments, activities, and mood over diverse timeframes (ie, weekly, monthly, and annually). This aids in providing users with a deeper understanding of their pain journey, ultimately supporting more effective self-management.

#### Wide Data Acquisition Using Smartphones

While large data sets on repeated multidimensional pain ratings beyond intensity alone are still few and far between, smartphones offer a unique opportunity to expand data acquisition beyond classical experimental settings [74]. Smartphone access has increased tremendously in the past decade (84% of US households reported owning at least 1 smartphone) [76]. Data acquired remotely through smartphone apps facilitate large-scale, real-world studies without the constraints of traditional laboratory studies. The results of such pragmatic studies are more generalizable than highly selective traditional randomized controlled trials [77-79]. SOMA*Science* was built to allow remote monitoring of longitudinal symptoms and treatments to maximize high-quality data in large-scale pragmatic studies. To further facilitate a much wider array of user input, the SOMA app is compatible with both Android and iOS devices, meaning anyone in the United States with a smartphone can use it.

Smartphone-based pain assessments offer a solution to the limited geographic, economic, and cultural diversity in traditional pain studies. Smartphones are pervasive, even in low-resource [80] or rural areas [72,81], where almost half of the world’s smartphone owners live [82]. They are also widely used by older adults [83], who are often left out of laboratory-based pain studies. Additionally, there is a need to consider how pain and its treatment vary across racial, ethnic, and cultural backgrounds for comprehensive care. Even within a specific culture, there are important differences in how pain is experienced and treated across different racial and ethnic backgrounds that need to be accounted for to deliver the best pain care [84-86]. Upcoming translations of the app into languages like Spanish and German, in collaboration with experts familiar with the culture, will further diversify data and insights. Translation into other languages will follow, and collaboration to translate the app is welcomed.

## Section 3: Data Content

### Overview

With the rising number of health-focused smartphone apps, there is also a growing need for transparency in the selection of measures for the app. Here we briefly detail the process of selection for each measure in SOMA*Science* as assessed through the SOMA app, its basis in the scientific and clinical literature, and what gaps it was chosen to address. The goal is to provide transparency in the design and selection process to facilitate the development of research studies using SOMA*Science*. Specific measures may be refined over time with user feedback and as scientific studies using SOMA*Science* identify areas for improvement.

### Measuring Pain Intensity, Unpleasantness, and Interference

**Figure 4:**
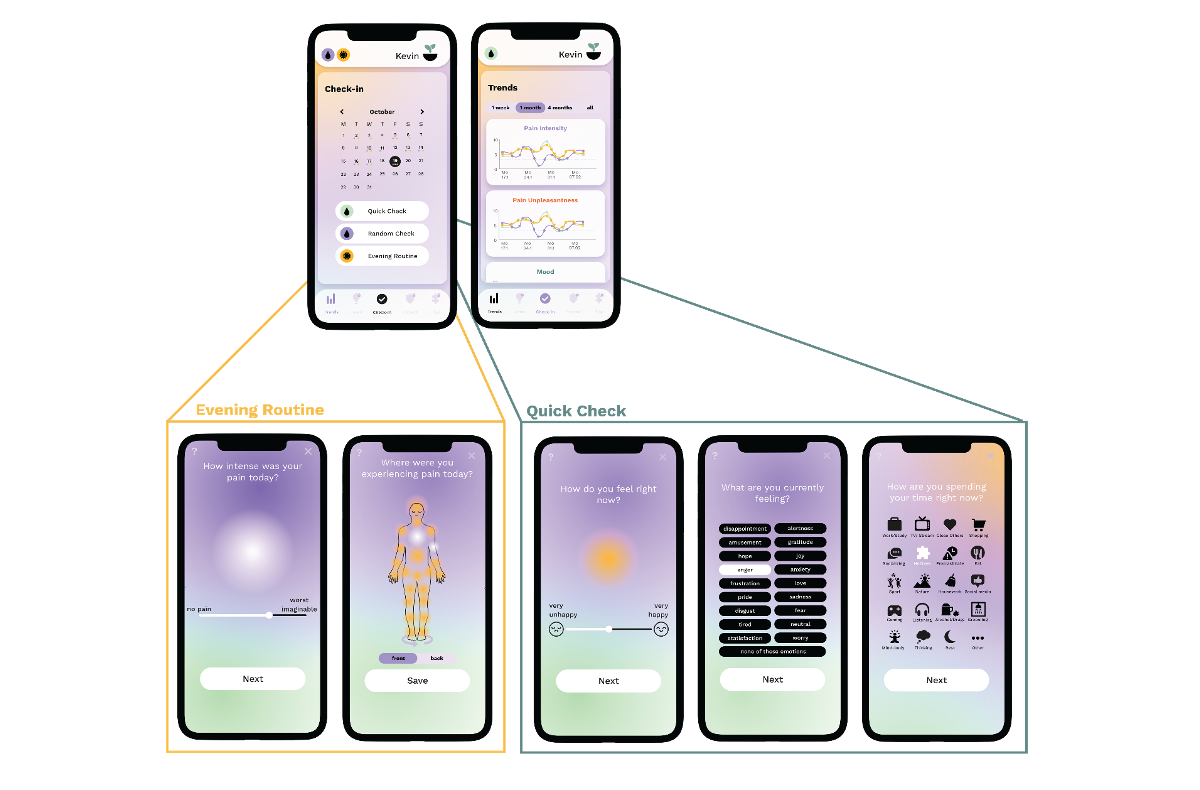
Upper panel: example screens on the SOMA app for check-ins and trends. Lower panel: example screens on the SOMA app from Quick Check, Random Check, and Evening Routine. From left to right: assessing pain on a visual analog scale (VAS), indicating pain location, assessing mood on a VAS, emotion selection, and activity selection.

Multidimensional pain assessment is a crucial aspect of clinical practice as it helps determine the effectiveness of treatment and recovery. The SOMA app assesses the pain intensity scale in addition to pain interference and pain unpleasantness to provide a more holistic measure of daily pain symptom load [87]. Participants are asked to rate each pain question on a sliding VAS, providing a simple and comparable assessment of daily pain experience (Figure 4). This operationalizes a practice advocated in the International Statistical Classification of Diseases and Related Health Problems for providers to use a composite rating of pain intensity, distress, and interference of pain to determine overall burden [88]. The composite pain score provides a multidimensional solution that balances the limitations of the unidimensional pain scale while still being easily quantifiable. The ability to analyze the 3 measures individually and as a composite score helps identify meaningful individual variability, enabling specific predictions between individuals. For example, pain unpleasantness could be more predictive of future pain in individual A, while pain interference may be more important for individual B’s long-term outcomes.

Importantly, SOMA’s 3 pain questions were chosen because they are directly comparable to results from established pain questionnaires, such as the Brief Pain Inventory [89] or the McGill Pain Inventory [90]. They also satisfy the standards set by major scientific and regulatory bodies, such as the IMMPACT recommendations, the NIH Helping to End Addiction Long-term initiative, and the FDA guidelines for assessing multidimensional components of pain [13,28,29,87]. In this way, SOMA’s multidimensional pain assessment of intensity, unpleasantness, and interference can provide important supplemental measures that are directly comparable to established clinical benchmarks and standards of care. This is critical for researchers looking to establish and validate novel pain biomarkers or end points.

### Measuring Pain Locations

Pain localization is an important aspect of pain assessment. Conventional methods of measuring pain location in medical appointments and research studies involve having individuals indicate it on a body map, such as the Brief Pain Inventory [89], the McGill Pain Questionnaire [91], or the Michigan Body Map [92]. This approach can pinpoint differences in peripheral and central pain pathology based on the localization and stability of pain representation over time. For instance, nociceptive or inflammatory pain is usually precisely localized somatically and does not change much over time, while neuropathic or chronic primary pain is often experienced in multiple bodily locations, radiates, or changes over time [93].

**Figure 5:**
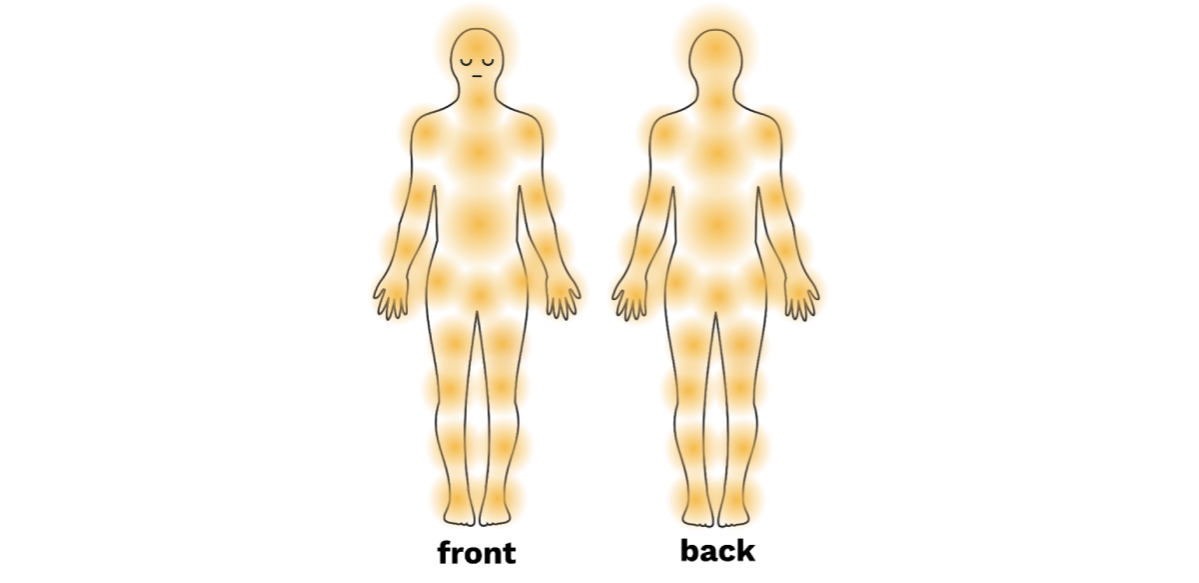
Pain map included on the SOMA app covering 46 discrete pain locations.

More recent methods of digital quantification, like the ones used on the SOMA app, have established the reliability and validity of body maps for pain assessments [94,95]. Interactive body maps delivered through digital or tablet apps are more effective than traditional paper or laptop assessments [92,96]. Yet a review of smartphone apps that use the body map for tracking pain found that few actually quantified the location ratings or provided any summary feedback [97]. The SOMA app’s interactive body map offers 46 different discrete location options on the front and back of the body that participants indicate in every daily check-in (Figure 5 and Multimedia Appendix 1). The use of discrete points ensures uniformity across devices and accounts for differences in participants’ finger size or dexterity. The “Trends” section of the SOMA app displays the body map with the percentage of times a location has been selected, enabling users to visualize the frequency of pain at a given location. For participants who experience nonspecific, difficult-to-localize, or widespread pain, such as fibromyalgia, there is an additional option to indicate “My pain is everywhere” on the body map.

### Measuring Interventions

The treatment of pain has been incredibly difficult to get right. The newest clinical guidelines advocate the use of multimodal, multidisciplinary approaches [27,98,99]. Such approaches emphasize a combination of pain treatments that include medications, restorative therapies (eg, physical therapy), interventional procedures (eg, epidural injections), behavioral interventions (eg, cognitive behavioral interventions), and complementary and integrative medicine (eg, acupuncture). Combinations of these therapies have been associated with the best long-term pain outcomes [100,101] and satisfy a biopsychosocial approach to pain [102].

It can be challenging for individuals and providers to determine which treatments are most effective for them, as the effects of many treatments for pain may not become apparent for weeks or even months (eg, cognitive and physical interventions, certain medications, and surgery) [103,104]. In determining how to measure treatments through the SOMA app, we followed the recommendations of the 2019 Department of Health and Human Services Pain Strategy [27].

In the case of medications, many different pathophysiologic mechanisms are targeted with different classes of medications. The use of different medications often changes over time, so we designed the medication screen on the SOMA app to be able to capture such changes. We, therefore, included 20 options across the main classes of pain medications for both acute and chronic pain on the SOMA app, detailed further in Multimedia Appendix 1.

A second treatment screen includes the recommended nonpharmacologic approaches to pain. Combinations of these treatments are often used by a single person over time to target different pain mechanisms [105]. SOMA*Science* currently provides the ability for people to track up to 20 different nonpharmacologic therapies across these 4 major classes, detailed in Multimedia Appendix 1. SOMA*Science*’s broad treatment tracking capabilities therefore facilitate the type of wide data needed to understand differences in treatment use across users.

### Measuring Emotions

Emotion and pain are fundamentally related. Definitions of pain acknowledge that pain is partially an emotional experience [106,107], yet few researchers would reduce pain down to just another emotional state such as sadness or happiness. The complexity of the pain-emotion relationship is highlighted by reviews of the neural circuits of each construct, showing both shared and functionally dissociable brain regions [108,109]. Unfortunately, the theoretical and empirical understanding of how emotion and pain are connected is limited, as much previous research only focuses on cross-sectional correlations between the 2 constructs [110,111].

Despite the renaissance of emotion research since the 1960s [112], emotion researchers continue to disagree on what constitutes an appropriate emotion measure [113]. The discrete emotion perspectives suggest that specific emotions such as anger, fear, happiness, sadness, disgust, and surprise are special kinds of biologically distinct responses associated with unique behavioral, physiological, and experiential correlates [114,115]. The dimensional perspectives consider emotions to be organized along a set of common dimensions such as valence (unpleasantness) and arousal (intensity) that are combined with cognitive and cultural knowledge to form emotional states.

When designing an emotion list for the SOMA app, we wanted a set of emotions that are commonly experienced, had a balance of positive and negative emotions, and were relevant to pain experiences (eg, worry). We started by selecting emotions experienced more than 5% of the time from a large-scale ecological momentary assessment study [116], removing rare emotions such as contempt or awe. Next, we balanced the remaining emotion set by using positive and negative emotions from the clinically relevant scale (Positive and Negative Affect Schedule) [117]. Lastly, we allowed participants to indicate no emotion (neutral) or emotion not listed (other).

A complementary method from a dimensional perspective is to assess general mood through a single-dimensional VAS of valence ranging from very unpleasant to very pleasant. Although there are other dimensional scales that could also be assessed, such as arousal [118,119] or goal congruence [120], valence is known to capture the majority of variance between emotion states [121], as self-reports of emotions tend to be highly correlated within a positive or negative valence [122]. Accordingly, on the SOMA app, we ask users to evaluate their current, past, or future mood on this VAS, ranging from unpleasant (0) to very pleasant (100). One major benefit of this measure is that we can quantify daily emotional experiences even if the participant does not self-report any discrete emotions from our finite list, reducing the need to interpolate or remove missing data from our analysis of the dynamics of emotion and pain (Figure 4).

### Measuring Activities

People’s experiences of both pain and emotion are intrinsically tied to the activities they are engaging in each day. There have been many large-scale studies investigating the relationship between emotions and behavior, showing that physical exercise is meaningfully associated with reduced mental health difficulties [123] and that people’s choice of activities is motivated by minimizing negative affect and maximizing positive affect [124,125]. In particular, people seem to engage in mood-elevating activities (eg, socializing) when they are feeling down and mood-depressing activities (eg, work and chores) when they are feeling up. At the same time, being in pain affects both mood and daily activities.

Most people intuitively reduce their activities when they experience new-onset pain. Acute pain generally functions as an alarm bell in the brain to signal tissue damage, with the urge to rest considered a protective mechanism to prevent further injury and promote healing [126]. However, outside of the initial acute phase, a lack of activity can hinder long-term recovery and may signal underlying changes in affective and motivational brain circuits that have been causally linked to the transition from subacute to chronic low back pain [126,127]. The synergistic impact of activity engagement on mood, pain, and physical function is why activity engagement is promoted by pain self-management and rehabilitation programs alike [128]. While many people assume that patients with chronic pain move less than those not in pain, objective actigraphy data do not differ between patients with chronic pain and no-pain controls [129,130]. What has been less studied is the types and range of daily activities and how they change between acute and chronic pain stages. While people may recognize changes in their daily activities as a result of pain (eg, no longer walking as much), they may not have insight into the relationship between pain, mood, emotion, and activities (ie, which activities increase or decrease pain in the short vs long term). It remains unclear whether certain patterns of activity engagement at different stages of pain experience are important for long-term outcomes.

For this reason, we added an activities-tracking feature alongside mood and pain tracking. The activities screen on the SOMA app contains 20 activities that are known to have a dynamic relationship with mood based on large-scale, longitudinal data sets [125] or to be common among patients with pain (eg, medical visits). Specific activities selected are detailed in Multimedia Appendix 1. In addition to having the person report either momentary or daily activities, we also have participants reflect on how each activity they completed made them feel that day and how much it affected their pain. Taken together, these measurements provide a full picture of participants’ daily activities and help understand the potential bidirectional relationships between emotion, pain, and behavior. Users are also able to visualize their ratings of how much a given activity affected their pain and mood over time in the dedicated trends screen, which may help people develop insight into how certain activities help or hinder their recovery.

### Measuring Predictions

Expectations play an undeniably large role in pain perception. In artificial laboratory settings where healthy participants receive painful stimulation, a wealth of findings show that expectations about pain can increase the pain experience (nocebo effect) or conversely decrease it (placebo effect) [131,132]. This has important implications for the experience of pathological pain [133,134], where expectations about pain treatment are reliable predictors of treatment response [135]. These studies suggest that an ongoing cognitive modulation of pain is an important determinant of ongoing pain perception [136]. Expectations for pain relief most likely shift over time, the longer someone has experienced pain the more difficult the pain is to control [137,138]. Subtle longitudinal changes in expectations are believed to occur as pain becomes chronic, but capturing these changes in research studies is challenging. For this reason, we included an assessment of daily predictions about pain, mood, and activities on the SOMA app to capture how predictions vary over time within the same person or between different types of users (eg, patients with acute vs chronic pain).

We decided to assess expectations on the SOMA app by asking users to predict their expected levels of pain intensity, unpleasantness, interference, mood, and activities for the next day using the same scales used to capture their actual rating for that day. This allows the assessment of the bilateral influence of pain and mood expectations on actual experiences of pain, mood, and activities that are entered the following day.

## Discussion

There is a great need for easy-to-use tools that help those in pain, their medical providers, and the larger health care system identify risk factors and predict the onset of chronic pain. Pain management is a rapidly evolving field that increasingly relies on assessments and treatments that are multimodal and multidisciplinary. Traditional, unidimensional assessments of self-reported pain fail to capture the nuances of pain experience and multimodal pain management. Therefore, there is an urgent need for research tools that have been specifically designed to capture this complexity.

To address this gap, we built the SOMA*Science* platform. Briefly, the platform uses the smartphone app SOMA to collect longitudinal, multidimensional, ESM-based pain data that capture daily pain intensity, unpleasantness, inference, mood, activities, and predictions. The SOMA app provides a simple and pleasing user interface that can promote pain self-management through visualization of pain trends over time, helping encourage individual insight into factors that exacerbate or alleviate pain. The visualizations provided can also be used to improve communication of multidimensional pain burdens to health care providers. At the same time, the larger SOMA*Science* platform enables user data to be included in registered single and multicenter studies.

In this paper, we have detailed the clinical and technological considerations taken into account in developing SOMA*Science* and the scientific rationale behind its measurements. We believe this platform is capable of meeting the requirement for tools to acquire deep and wide-ranging pain data over time, which has been largely absent from existing pain data sets. As such, SOMA*Science* can be used to answer a broad range of research questions, such as the correlation between initial pain dynamics and the eventual development of chronic pain (ie, predicting the transition from acute to chronic pain), evaluating both short-term and long-term effects of various treatments on pain experiences, or identifying distinct symptom clusters (ie, pain phenotypes). Moreover, the data available are sufficient to calculate more detailed multidimensional and longitudinal clinical trial or observational study end points.

The primary focus of this paper was to introduce SOMA*Science* as a platform for scientific studies. In the long term, we also plan to build platforms with a more clinical focus that facilitate integration with medical care (SOMA*Clinic*) and the support of treatments (SOMA*Therapeutics*). This will involve connecting the SOMA app to electronic medical records and including interfaces to health trackers (eg, daily actigraphy, heart rate, or sleep data from health kits or wearables). The intention is to have a significant positive impact, both in terms of advancing research on pain and improving the lives of people with pain.

## Appendix

Multimedia Appendix 1 The technological and design details regarding the building of the SOMA app, as well as specific measures selected for SOMA app screens, that may be of interest to some readers.

## Abbreviations

APA: American Psychiatric Association
ESM: experience sampling methodology
FDA: Food and Drug Administration
IMMPACT: Initiative on Methods, Measurement, and Pain Assessment in Clinical Trials
mHealth: mobile health
NIH: National Institutes of Health
VAS: visual analog scale
