# Supplementary material for: SOMAScience: A Novel Platform for Multidimensional, Longitudinal Pain Assessment
Source: JMIR Mhealth Uhealth. 2024 Jan 12;12:e47177. doi: 10.2196/47177 (PMC10818247; doi:10.2196/47177)
Supplement: Multimedia Appendix 1 [file mhealth_v12i1e47177_app1.docx]

Supplemental Information for SOMA*Science*: A novel platform for multidimensional, longitudinal pain assessment

### Technological development

####

#### REST API and Database

We started development of the SOMA App using mindLAMP [135,136], a versatile open-source app development platform offered by the Digital Psychiatry Lab at Beth Israel Deaconess Medical Center (BIDMC). We ultimately chose to move away from mindLAMP Bbecause of the level of customization required to support SOMA*Science* and the effort involved in customizing the visual design, . Instead we invested in a custom REST Application Programming Interface (API) backed by MongoDB [137]. Writing our own API gave us the opportunity to focus software engineering efforts on building a secure and robust platform for data access. The schema-less nature of MongoDB provided us with the flexibility needed to store different SOMA*Science* data.

####

#### Design and Frontend

Users have a strong preference to interact with aesthetically pleasing environments [138]. Well-designed apps receive larger traction, are more likely to be recommended and are perceived as more professional [139,140]. For that reason we took great care to develop a well-designed interface with support from designers at the Rhode Island School of Art and Design. The design was first drafted in Adobe XD, an industry standard design tool for web and mobile applications. Some of the app-specific design elements include a distinct visual identity (see Figure 4), an app tour to orient users with the main functionalities, user-friendly button sizes, easy navigation, automated help texts that appear once a user gets stuck throughout a check-in an onboarding chat, and an onboarding chat for early orientation.

The frontend of our application is built with React Native [141], a cross-platform user-interface (UI) framework. This framework leverages React, a popular web application library, and abstracts the user interface rendering to allow support for devices including Apple and Android smartphones, traditional web, smart TVs and more. React Native Elements, a user interface library, provided low level UI primitives like headers and buttons.

To facilitate the developer experience we chose Expo to manage our React Native application. Expo is a collection of tools and software libraries that facilitate the development of React Native applications. We use Expo Application Services (EAS) to help automate compiling the SOMA App for both Android and iOS, and we use the Expo Software Development Kit (SDK) to rapidly develop common app functions like splash screens, navigation, and notifications.

### Response bias minimization

Several ESM assessments on the SOMA App were designed to minimize response biases for scientific use. One common challenge in analyzing public app or user data is the potential for response biases due to the structure of the screens. Research has shown that choices located at the natural reading direction (e.g. the upper left or right corner of a screen) may be favored [78]. To address this issue, our application randomizes the order of emotion labels and activities across users and maintains that order for each user. This randomization is a compromise between the user’s expectation of consistent button location versus the scientific need to reduce bias across users.

Another issue that can arise with linear rating scales is the tendency for users to select default settings to save time and effort. To mitigate this, all VAS (Visual Analog Scale) assessments on the SOMA App display a default starting point in the middle of the screen as a reference for zero, but then require users to make at least one selection before they can advance to the next screen, thus reducing the chances of default responding.

### Maximizing Privacy and Security

Recent reviews suggest that a large number of mobile health applications contain significant safety concerns, often related to the quality of information presented or to the security of participants’ data [142].

We implement technical and organizational measures in an effort to protect data from loss, theft, misuse and unauthorized access, disclosure, alteration and destruction, taking into account the nature of the data that we process and risks associated with special categories of data we collect (e.g., information about health). These measures include Pseudonymization and tokenization of certain categories and encryption of data in transit via SSL and in rest via block storage level encryption. Currently, SOMA App data is stored on password and multifactor authentication protected Brown University servers. These Brown University servers are located on a separate network requiring the use of VPN to access them.

In accordance with European privacy rights, users have rights to their data. This includes the right to access, restrict use, correct and delete their data as detailed in the Privacy Policy [143]. In compliance with Apple's Account Deletion and Data Deletion guidelines, we provide both Android and Apple users the clear option to delete their account and data through the click of a button.

To protect privacy and security, SOMA only uses de-identified or aggregated data, which cannot be associated with an individual. Individuals taking part in dedicated studies might provide more detailed information. However, this information is not acquired or stored on SOMA but will be acquired and handled independently by the individual research institution conducting the study.

Individual SOMA App Measures:

The following include the exact selections for measures on the SOMA app across the following categories:

*Pain Locations:*

**Pain Map included on the SOMA App covering 46 discrete pain locations***:* front head, front neck, front chest, front shoulder left, front shoulder right, front upper arm left, front upper arm right, front lower arm left, front lower arm right, front hand left, front hand right, front abdomen, front hip left, front hip right, front genitals, front upper leg left, front upper leg right, front knee left, front knee right, front lower leg left, front lower leg right, front feet left, front feet right, back head, back neck, back chest, back shoulder left, back shoulder right, back upper arm left, back upper arm right, back lower arm left, back lower arm right, back hand left, back hand right, back, lower back, back hip left, back hip right, back genitals, back upper leg left, back upper leg right, back knee left, back knee right, back lower leg left, back lower leg right, back feet left, back feet right.

*Pain Medications:*

The following 20 options across the main classes of pain medications for both acute and chronic pain are included on the SOMA App: Muscle relaxants, Lidocaine patch/cream, Corticosteroids, Aspirin, Naproxen/Ibuprofen/Diclofenac, Celecoxib, Acetaminophen, Gabapentin/Pregabalin, Tricyclic Antidepressants, SNRI, SSRI, Hydro/oxycodone, Tramadol/Tapentadol, Morphine, Codeine, Fentanyl, Opioid Agonist Treatments, Cannabis, Ketamine, and other. For options that include a broad category of medications, such as ‘muscle relaxants’ or ‘SNRI’, users are able to consult a help section that lists all generic and name brand forms of the drug since they may not know their specific medication falls in that category. For options that include a specific type of drug in a category (eg celecoxib as an NSAID), the help text lists name brand forms of the drug.

*Non-pharmacological pain treatments:*

The restorative therapies assessed are Dry Needling/Trigger Point Injections, Massage, Physical Therapy, TENS unit, Heat/Ice, Chiropractic/Osteopathic manipulation. Interventional procedures include Epidural injections, Nerve block, Radio-frequency Ablation, Surgery, Spinal Cord Trial/Implant. The measured behavioral interventions are Psychotherapy/Counseling, Biofeedback, and Transcranial Magnetic Stimulation. The measured complementary and integrative therapies are Acupuncture/Cupping, Meditation, Tai Chi/Yoga, Hypnosis, Herbs/Supplements, as well as An ‘Other’ option for anything not captured.

*Activities:*

The 20 activities included are: social media, thinking, TV/stream, socializing, medical visit, hobbies, work/study, rest, mindfulness, sport, nature, listening, eat, housework, shopping, gaming, close others, grooming, alcohol/drugs, and other.

*Emotions:*

The 20 emotions included in the SOMA app are: alertness, amusement, anger, anxiety, disappointment, disgust, fear, frustration, gratitude, hope, joy, love, neutral, other, pride, sadness, satisfaction, tired, worry.

References:

[135] “mindLAMP Digital App.” [Online]. Available: https://www.digitalpsych.org/lamp.html

[136] “LAMP Platform.” [Online]. Available: https://docs.lamp.digital/

[137] “MongoDB,” Developer Data Platform. [Online]. Available: https://www.mongodb.com/

[138] T. Kato, “Functional value vs emotional value: A comparative study of the values that contribute to a preference for a corporate brand,” *Int. J. Inf. Manag. Data Insights*, vol. 1, no. 2, p. 100024, Nov. 2021, doi: 10.1016/j.jjimei.2021.100024.

[139] A. J. Lazard, J. S. Babwah Brennen, and S. P. Belina, “App Designs and Interactive Features to Increase mHealth Adoption: User Expectation Survey and Experiment,” *JMIR MHealth UHealth*, vol. 9, no. 11, p. e29815, Nov. 2021, doi: 10.2196/29815.

[140] G. H. Iten, A. Troendle, and K. Opwis, “Aesthetics in Context—The Role of Aesthetics and Usage Mode for a Website’s Success,” *Interact. Comput.*, vol. 30, no. 2, pp. 133–149, Mar. 2018, doi: 10.1093/iwc/iwy002.

[141] “ReactNative.” [Online]. Available: https://reactnative.dev/

[78] S. Hoober and E. Berkman, *Designing mobile interfaces: Steven Hoober, Eric Berkman*, First edition. Sebastopol, CA: O’Reilly, 2012.

[142] S. Akbar, E. Coiera, and F. Magrabi, “Safety concerns with consumer-facing mobile health applications and their consequences: a scoping review,” *J. Am. Med. Inform. Assoc.*, vol. 27, no. 2, 2020, [Online]. Available: https://academic.oup.com/jamia/article/27/2/330/5585394

[143] “SOMA App Privacy Policy.” [Online]. Available: https://somatheapp.com/privacy-policy-2/
